# LRRK1 is critical in the regulation of B-cell responses and CARMA1-dependent NF-κB activation

**DOI:** 10.1038/srep25738

**Published:** 2016-05-11

**Authors:** Keiko Morimoto, Yoshihiro Baba, Hisaaki Shinohara, Sujin Kang, Satoshi Nojima, Tetsuya Kimura, Daisuke Ito, Yuji Yoshida, Yohei Maeda, Hana Sarashina-Kida, Masayuki Nishide, Takashi Hosokawa, Yasuhiro Kato, Yoshitomo Hayama, Yuhei Kinehara, Tatsusada Okuno, Hyota Takamatsu, Toru Hirano, Yoshihito Shima, Masashi Narazaki, Tomohiro Kurosaki, Toshihiko Toyofuku, Atsushi Kumanogoh

**Affiliations:** 1 Department of Immunopathology, World Premier International (WPI) Immunology Frontier Research Center, Osaka University, Suita, Osaka 565-0871, Japan; 2 Department of Respiratory Medicine, Allergy and Rheumatic Disease, Graduate School of Medicine, Osaka University, Suita, Osaka 565-0871, Japan; 3 The Japan Agency for Medical Research and Development - Core Research for Evolutional Science and Technology (AMED-CREST), Tokyo, Japan; 4 Laboratory for Lymphocyte Differentiation, WPI Immunology Frontier Research Center, Osaka University, Suita, Osaka 565-0871, Japan; 5 Laboratory for Lymphocyte Differentiation, RIKEN Center for Integrative Medical Sciences (IMS), Yokohama, Kanagawa 230-0045, Japan; 6 Laboratory for Integrated Cellular Systems, RIKEN Center for Integrative Medical Sciences (IMS-RCAI), Yokohama, Kanagawa 230-0045, Japan; 7 Department of Clinical Application of Biologics, Graduate School of Medicine, Osaka University, Suita, Osaka 565-0871, Japan; 8 Department of Pathology, Graduate School of Medicine, Osaka University, Suita, Osaka 565-0871, Japan; 9 Department of Geriatric Medicine and Nephrology, Graduate School of Medicine, Osaka University, Suita, Osaka 565-0871, Japan; 10 Department of Otorhinolaryngology-Head and Neck Surgery, Graduate School of Medicine, Osaka University, Suita, Osaka 565-0871, Japan; 11 Department of Neurology, Graduate School of Medicine, Osaka University, Suita, Osaka 565-0871, Japan; 12 Department of Immunology and Regenerative Medicine, Graduate School of Medicine, Osaka University, Suita, Osaka 565-0871, Japan

## Abstract

B-cell receptor (BCR) signaling plays a critical role in B-cell activation and humoral immunity. In this study, we discovered a critical function of leucine-rich repeat kinase 1 (LRRK1) in BCR-mediated immune responses. *Lrrk1*^*−/−*^ mice exhibited altered B1a-cell development and basal immunoglobulin production. In addition, these mice failed to produce IgG3 antibody in response to T cell–independent type 2 antigen due to defects in IgG3 class-switch recombination. Concomitantly, B cells lacking LRRK1 exhibited a profound defect in proliferation and survival upon BCR stimulation, which correlated with impaired BCR-mediated NF-κB activation and reduced expression of NF-κB target genes including Bcl-x_L_, cyclin D2, and NFATc1/αA. Furthermore, LRRK1 physically interacted and potently synergized with CARMA1 to enhance NF-κB activation. Our results reveal a critical role of LRRK1 in NF-κB signaling in B cells and the humoral immune response.

B cells play central roles in humoral immune responses. Antibodies resulting from B cell activation serve to eliminate pathogens and thereby protect the host from viral, bacterial, and parasitic infections^1^. B-cell responses fall into two types, based on the requirement for T-cell help in antibody production^2^: T cell–dependent (TD) or T cell–independent (TI). TD antigens are captured by B-cell receptor (BCR) and presented to cognate helper T cells on MHC class II molecules^3^. On the other hand, T cell–independent type 2 (TI-2) antigens, of which polysaccharides are representative, crosslink the BCR and elicit antigen-specific antibody responses^4^. This feature distinguishes TI-2 antigens from T cell–independent type 1 (TI-1) antigens such as lipopolysaccharide (LPS), which induce polyclonal B-cell activation. The specific recognition of antigens through the BCR initiates intracellular signaling that is required for B-cell activation, antigen presentation, and development^5^. Engagement of the BCR induces phosphorylation of tyrosine residues in the immunoreceptor tyrosine-based activation motifs of Igα and Igβ by Lyn, a Src family kinase. Subsequently, multiple signaling components including protein tyrosine kinases such as Syk and Btk and their adaptor molecules are recruited to the BCR, eventually leading to the activation of phospholipase Cγ2 (PLCγ2). Activated PLCγ2 generates two second-messenger products: the membrane lipid diacylglycerol (DAG) and the soluble inositol-1,4,5,-trisphosphate (IP_3_), which coordinately induce Ca^2+^ flux and activate the NFAT/NF-κB/mitogen-activated protein kinase (MAPK) cascade to regulate B-cell development and activation^6^.

NF-κB plays a crucial role in humoral immunity through a variety of BCR-mediated responses including B-cell activation, proliferation, survival, and effector functions^7^. Moreover, dysregulation of the NF-κB pathway can contribute to B-cell lymphomagenesis^8^,^9^. A hallmark of the activated B-cell subtype of diffuse large B-cell lymphoma (ABC-DLBCL) is constitutive NF-κB activation due to chronic active BCR signaling^10^. B-cell lymphomas in which NF-κB signaling pathways are constitutively activated have been also described in mantle cell lymphoma and mucosa-associated lymphoid tissue lymphoma^11^. Therefore, the mechanisms that properly regulate NF-κB function are clinically quite important. BCR-induced NF-κB activation is governed by the CBM complex, which contains CARMA1 (caspase recruitment domain, CARD, membrane-associated guanylate kinase, MAGUK, protein 1), BCL10 (B-cell lymphoma 10), and MALT1 (mucosa-associated lymphoid tissue lymphoma translocation protein 1)^12^. Formation of this complex is triggered by phosphorylation of CARMA1 by protein kinase C-β (PKC-β), which allows CARMA1 to recruit BCL10 and MALT1 into cellular membranes^13^. BCL10 and MALT1 then activate the IKK complex, which phosphorylates IκB (an inhibitor of NF-κB), resulting in its destruction and ultimately leading to activation of NF-κB. Although CARMA1 functions as an essential scaffolding platform for the BCR-dependent NF-κB signaling pathway, its regulatory mechanism has not been fully elucidated.

Leucine-rich repeat kinase 1 (LRRK1) belongs to a member of the ROCO family of proteins, which have multiple functional domains including ankyrin-like repeats, leucine-rich repeats (LRRs), a Ras-like GTPase domain (ROC) and an adjacent C-terminal domain (COR), and a serine–threonine kinase domain. Its homolog LRRK2 shares most domains with LRRK1 and has an additional LRRK2-specific repeat at the N-terminus. *LRRK2* is mutated in Parkinson’s disease (PD)^14^,^15^, as well as Crohn’s disease^16^. Despite its structural similarity with LRRK2, LRRK1 has distinct functions. For example, LRRK1 participates in intracellular trafficking of epidermal growth factor receptor (EGFR) in the cytosol^17^ and controls the orientation of mitotic spindles by regulating microtubule nucleation in the nucleus^18^. Furthermore, LRRK1 regulates autophagy^19^ and osteoclast differentiation^20^ under stress and physiological conditions, respectively. In addition, LRRK1 may contribute to tumorigenesis because of its ability to promote cell proliferation^21^ and interact with BCR-ABL1^22^, which exhibits elevated tyrosine kinase activity in lymphomas. Although LRRK1 has been shown to have a wide variety of functions and be expressed predominantly in B cells and monocytes in human peripheral blood^23^, its contribution to the immune system remains to be determined.

In this study, we found that murine B cells express *Lrrk1* over the course of their development. Therefore, we investigated the physiological role of LRRK1 in the humoral immune response and the molecular mechanism underlying this association. Mice lacking LRRK1 exhibited defects in B1a-cell development in the peritoneal cavity and the IgG3 antibody response to TI-2 antigen, yet they responded normally to TD antigen. Upon *in vivo* challenge with TI-2 antigen, *Lrrk1*^*−/−*^ B cells failed to induce the expression of activation-induced cytidine deaminase (AID) and IgG3 class-switch recombination (CSR). We also found that LRRK1 critically regulates BCR-induced B-cell proliferation and survival, as well as CARMA1-dependent NF-κB activation. Our data provide genetic evidence for the importance of LRRK1 in humoral immunity and suggest that LRRK1 may function as a novel modulator of CARMA1 to activate the NF-κB cascade.

## Results

### B cell development in *Lrrk1* ^*−/−*^ mice

**Figure 1:**
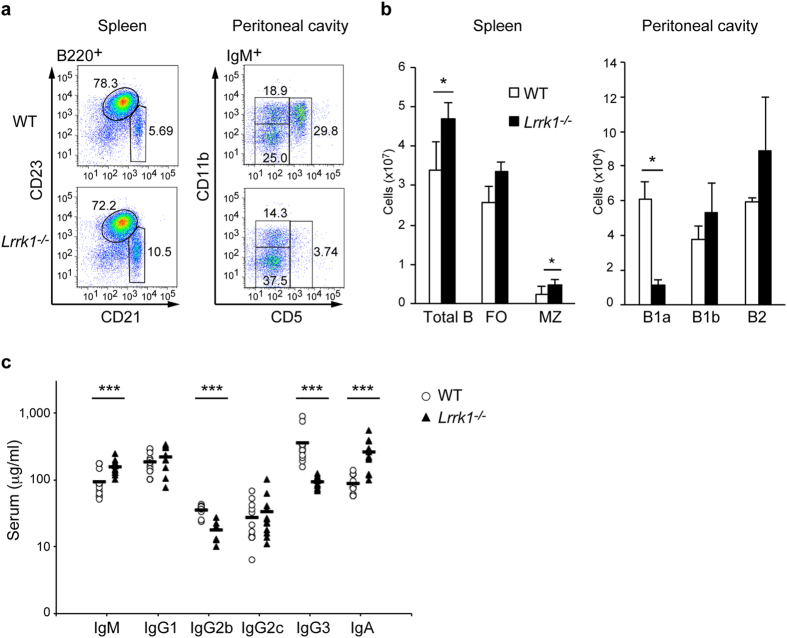
B-cell development and antibody production in *Lrrk1*^*−/−*^-deficient mice. (**a,b**) Flow cytometry of B-cell populations obtained from spleen and peritoneal cavity from wild-type (WT) and *Lrrk*^*−/−*^ mice. B-cell subsets were gated as follows: spleen, total B cell (B220^+^), follicular (FO; B220^+^ CD21^lo^CD23^hi^), marginal zone (MZ; B220^+^ CD21^hi^CD23^lo^); peritoneal cavity, B1a (IgM^+^ CD5^+^), B1b (IgM^+^ CD5^−^CD11b^+^), and B2 (IgM^+^ CD5^−^CD11b^−^). The number of cells in each subset was calculated on the basis of total cell count and flow-cytometric analysis. Data are representative of three independent experiments (**a**). Data are presented as means ± s.d. from three independent experiments (**b**). (**c**) ELISA of basal immunoglobulin titers in sera from wild-type and *Lrrk1*^*−/−*^ mice. Each symbol represents an individual mouse; small horizontal bars indicate mean values. There were 7–12 mice in each group. *P < 0.05, ***P < 0.001 (two-tailed unpaired Student’s *t*-test).

LRRK1 is expressed in B cells over the course of their development (Supplementary Fig. S1). To determine the physiological role of LRRK1 in B cells, we examined B-cell development in the bone marrow (BM), spleen, and peritoneal cavity of *Lrrk1*^*−/−*^ mice. Although the number of early B-lineage cells in BM in *Lrrk1*^*−/−*^ mice was reduced due to osteopetrosis, as previously described^20^, the percentages of pro-, pre-, immature, and mature recirculating B cells were similar to those of wild-type control mice (Supplementary Table S1). By contrast, *Lrrk1*^*−/−*^ mice had a higher total number of B cells in the spleen. Stains of splenic B cells for CD21 and CD23 revealed an increase in the number of marginal zone (MZ) B cells (CD21^hi^CD23^lo^) in *Lrrk1*^*−/−*^ mice relative to wild-type mice (Fig. 1a,b). Furthermore, *Lrrk1*^*−/−*^ mice had a much lower number and percentage of peritoneal B1a (IgM^+^ CD5^+^) cells, but not B1b (IgM^+^ CD5^−^CD11b^+^) or B2 (IgM^+^ CD5^−^CD11b^−^) cells (Fig. 1a,b). We also investigated basal immunological status in unimmunized mice. Serum titers of IgM and IgA were slightly higher in *Lrrk1*^*−/−*^ mice, whereas the amounts of IgG2b and IgG3 in *Lrrk1*^*−/−*^ mice were significantly reduced (Fig. 1c). These data indicate that LRRK1 is required for normal B-cell development, especially in B1a cells within the peritoneal cavity, as well as basal immunoglobulin production.

### Impaired T cell–independent IgG3 antibody response in *Lrrk1* ^*−/−*^ mice

**Figure 2:**
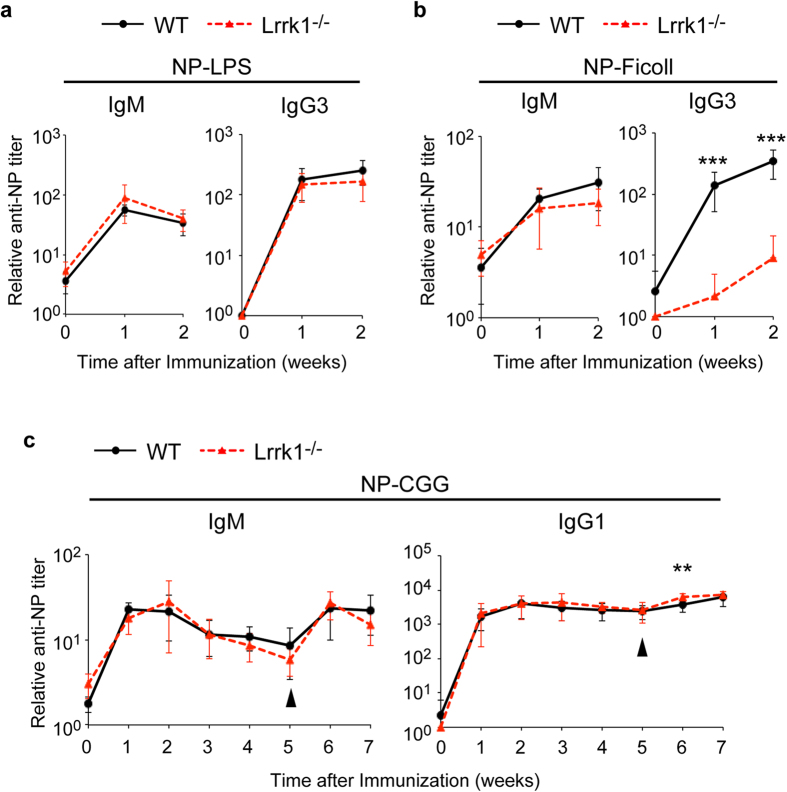
LRRK1 is indispensable for IgG3 production upon TI-2 stimulation. (**a,b**) IgM and IgG3 NP-specific antibody responses of wild-type and *Lrrk1*^*−/−*^ mice after immunization with NP-LPS (**a**) or NP-Ficoll (**b**), as assessed by NP-specific ELISA. **(c)** IgM and IgG1 NP-specific antibody responses of wild-type and *Lrrk1*^*−/−*^ mice after immunization with NP-CGG, boosted with NP-CGG 5 weeks after primary immunization, as assessed by NP-specific ELISA. Arrowheads indicate the day of secondary immunization. Data are presented as means ± s.d. for 7–9 mice for each genotype at each time point. **P < 0.01, ***P < 0.001 (two-tailed unpaired Student’s *t*-test).

To analyze the impact of LRRK1 deficiency on *in vivo* antibody responses, we immunized mice with NP-LPS, a representative TI-1 antigen, and NP-Ficoll, a TI-2 antigen. We detected similar amounts of anti-NP IgM and IgG3 antibodies against NP-LPS in both wild-type mice and *Lrrk1*^*−/−*^ mice (Fig. 2a). By contrast, when mice were immunized with NP-Ficoll, we observed a severe defect in production of IgG3 in *Lrrk1*^*−/−*^ mice (Fig. 2b); slight, but statistically insignificant decreases in IgM levels were also detected. To analyze the TD antibody response in *Lrrk1*^*−/−*^ mice, we immunized them with 4-hydroxy-3-nitrophenylacetyl-chicken-gammma-globulin conjugates (NP-CGG) and monitored the production of antigen-specific IgM and IgG1 antibodies. *Lrrk1*^*−/−*^ mice mounted primary immune responses comparable to those of wild-type mice (Fig. 2c). After NP-CGG re-challenge, anti-NP IgM and IgG1 production was also within the normal range, indicating that ablation of the LRRK1 protein does not affect production of primary and memory responses. Therefore, we conclude that LRRK1 is essential for IgG3 antibody production in the response to TI-2 antigens.

### LRRK1 is required for TI-2–dependent class-switch recombination

**Figure 3:**
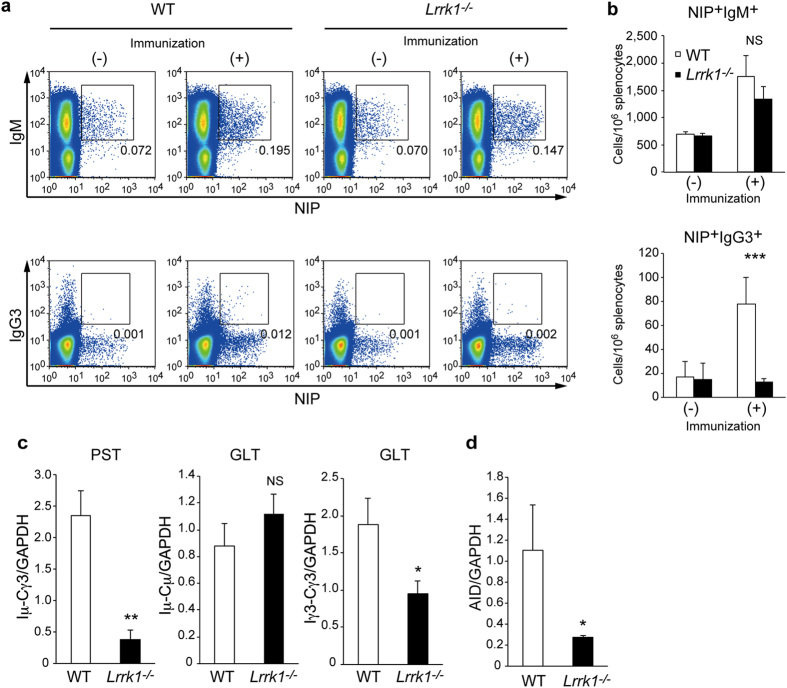
LRRK1 is required for IgG3 germline transcription and AID expression in response to TI-2 antigen. (**a**) NP-binding B cells from spleen of wild-type and *Lrrk1*^*−/−*^ mice 4 days after immunization with NP-Ficoll were detected by flow cytometric staining with NIP-APC. (**b**) NP-binding B cells per 10^6^ splenocytes were counted based on (**a**). Data are presented as means ± s.d. for 5–6 mice. (**c,d**) Quantitative RT-PCR analysis of post-switch transcript (PST) of the μ chain I region and γ3-chain C region (Iμ-Cγ3), gremline transcripts (GLT) (Iμ-Cμ, Iγ3-Cγ3) (**c**), and AID (**d**) expression in CD19^+^ B cells 4 days after immunization with NP-Ficoll. Data are presented as means ± s.d. of two independent experiments. NS, not significant. *P < 0.05, **P < 0.01, ***P < 0.001 (two-tailed unpaired Student’s *t*-test).

Because splenic MZ B cells rapidly and preferentially respond to TI-2 antigens to generate IgM and switched IgG antibodies, defects in TI-2–induced immunoglobulin class switch are often correlated with a numerical decrease or functional impairment of MZ B cells. *Lrrk1*^*−/−*^ mice had sufficient numbers of MZ B cells, so we investigated whether antigen-specific B cells could respond to TI-2 antigen and elicit class-switch recombination (CSR) in the absence of LRRK1. To this end, we measured the frequency of NP-specific B cells in the spleen 4 days after the NP-Ficoll immunization. Wild-type mice exhibited a significant increase in NP-specific IgM^+^ and class-switched IgG3^+^ B cells, as assessed by flow cytometory with 4-hydroxy-5-indo-3-nitrophenyl acetyl (NIP)-APC (Fig. 3a,b). By contrast, *Lrrk1*^*−/−*^ mice failed to generate NP-specific IgG3^+^ B cells and also exhibited a slight but statistically insignificant decrease in the number of NP-specific IgM^+^ cells (Fig. 3a,b).

The mechanism of CSR requires the expression of AID and germline transcription from I-region promoters upstream of each switch region, which allows AID to access switch-region DNA^24^. To further characterize the defect in antigen-specific IgG3^+^ B-cell generation from NP-Ficoll–immunized mice, we analyzed germline transcripts (GLTs) and post-switch transcripts (PSTs) encoding the μ-chain I region and γ3-chain C regions (Iμ-Cγ3). IgG3 mRNA was much less abundant in B cells from *Lrrk1*^*−/−*^ mice than in those from wild-type mice, as determined from the amounts of Iμ-Cγ3 PST (Fig. 3c). Furthermore, the level of GLTs initiated from the promoter of the γ3-chain I region (Iγ3-Cγ3) was significantly reduced in B cells from *Lrrk1*^*−/−*^ mice despite a similar abundance of GLTs from the μ-chain I-region gene (Iμ-Cμ) (Fig. 3c). Moreover, the level of AID mRNA in *Lrrk1*^*−/−*^ B cells was approximately 25% of that in wild-type B cells (Fig. 3d), suggesting that reduced AID expression may also contribute to the CSR defect in *Lrrk1*^*−/−*^ mice. Collectively, these results suggest that LRRK1 plays an essential role in the IgG3 antibody response to TI-2 antigen by regulating IgG3 GLT and AID expression.

### BCR-induced B-cell proliferation and survival requires LRRK1

**Figure 4:**
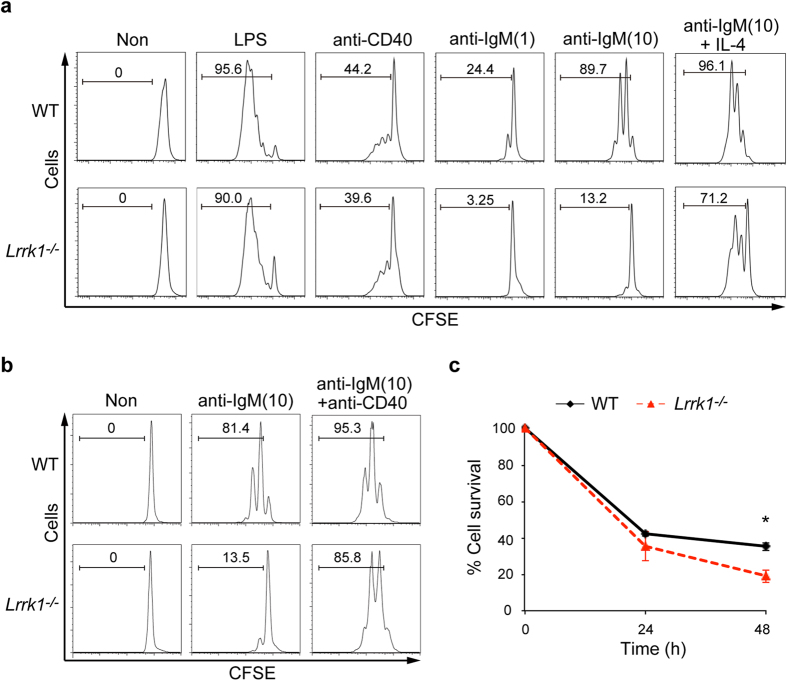
Impaired proliferation and survival upon BCR ligation of *Lrrk1*^*−/−*^ B cells. **(a,b**) Proliferation of wild-type and *Lrrk1*^*−/−*^ B cells stimulated for 72 h with LPS (10 μg/ml), anti-CD40 (10 μg/ml), or anti-IgM (1 or 10 μg/ml) (**a**) or with a combination of anti-IgM (10 μg/ml) and either IL-4 (5 ng/ml) (**a**) or anti-CD40 (10 μg/ml) (**b**). Proliferation of B cells was assessed by CFSE dilution by flow cytometry. Data are representative of three experiments (**a**) or two experiments (**b**). **(c)** Survival of wild-type and *Lrrk1*^*−/−*^ B cells cultured with 10 μg/ml anti-IgM, counted with Trypan blue; counts are presented as percentages relative to the initial cell number (defined as 100%). Data are presented as means ± s.e.m. Results were pooled from three independent experiments. *P < 0.05 (two-tailed unpaired Student’s *t*-test).

*In vivo* analysis of TI-2 immune responses in *Lrrk1*^*−/−*^ mice suggested that B cells lacking LRRK1 could be hyporesponsive. To test this idea and determine whether this phenomenon is due to an intrinsic abnormality of the mutant B cells, we examined the *in vitro* responses of wild-type and *Lrrk1*^*−/−*^ B cells upon stimulation with several mitogenic stimuli. In these experiments, we labeled purified B cells with carboxyfluorescein diacetate succinimidyl ester (CFSE), and monitored cell division by CFSE dilution. *Lrrk1*^*−/−*^ B cells exhibited impaired proliferation in response to anti-IgM stimulation (Fig. 4a). On the other hand, ablation of LRRK1 in B cells did not alter their proliferation in response to LPS or anti-CD40 stimulation. We also characterized the proliferative response to co-stimulation of anti-IgM with IL-4 or anti-CD40 (Fig. 4a,b). The defective response in BCR-stimulated *Lrrk1*^*−/−*^ B cells was significantly rescued by addition of IL-4 or anti-CD40, suggesting that signal evoked by IL-4 or anti-CD40 can compensate for LRRK1 deficiency after BCR stimulation. In accordance with the proliferation data, B cells lacking LRRK1 exhibited diminished survival following BCR ligation (Fig. 4c). It should be noted that survival rate of unstimulated B cells lacking LRRK1 is comparable to that of wild-type B cells (Supplementary Fig. S2a,b). These results suggest that LRRK1 signaling is essential specifically for BCR-mediated B-cell proliferation and survival.

### LRRK1 mediates BCR-dependent NF-κB activation

**Figure 5:**
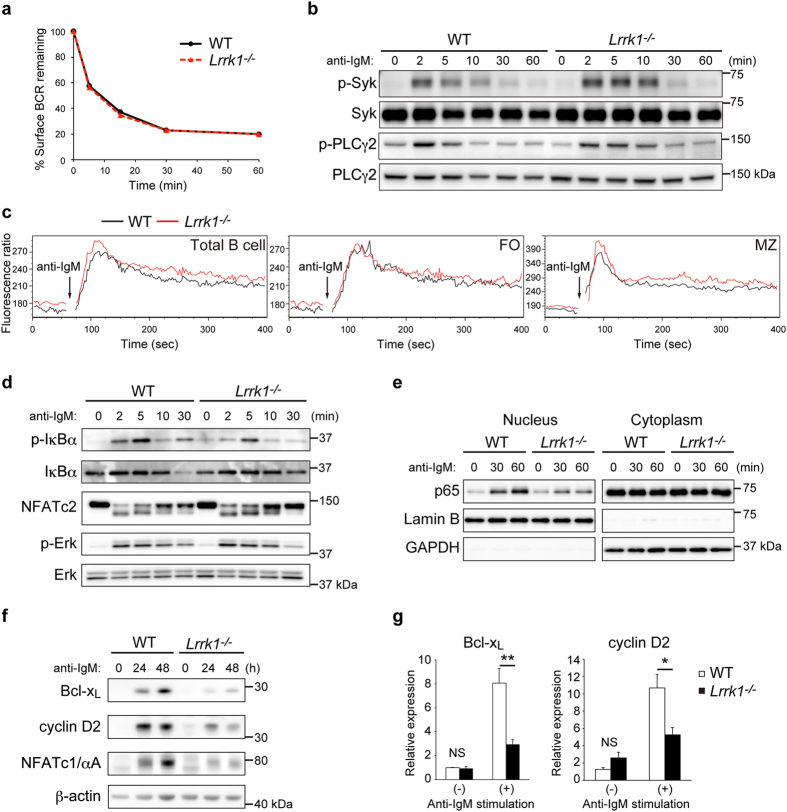
LRRK1 mediates BCR-dependent NF-κB activation. **(a)** BCR endocytosis of wild-type and *Lrrk1*^*−/−*^ mice, monitored by flow cytometry at the indicated times after staining with biotinylated anti-IgM. Cells were incubated for 30 min on ice in the presence of biotinylated anti-IgM, and then incubated at 37 °C for 0, 5, 15, 30, or 60 min. The level of BCR remaining on the cell surface is presented as mean fluorescence intensity relative to that of the 0 min sample (defined as 100%). Results are representative of three similar experiments. **(b)** Phosphorylation status of Syk and PLCγ2 of wild-type and *Lrrk1*^*−/−*^ B cells assessed by immunoblotting of whole-cell lysates obtained from B cells stimulated with anti-IgM. Data are representative of at least two independent experiments. **(c)** Ca^2+^ mobilization in response to stimulation with 10 μg/ml anti-IgM in wild-type and *Lrrk1*^*−/−*^ splenocytes stained with anti-B220 (total B cell), anti-CD21, and anti-CD23 (FO, B220^+^ CD21^lo^CD23^hi^; MZ, B220^+^ CD21^hi^CD23^lo^). Ca^2+^ flux was monitored by Indo-1AM imaging in the presence of 2 mM extracellular Ca^2+^, and all values are plotted as the FL5/FL4 fluorescence ratio (FL4 = 500–520 nm; FL5 = 400–420 nm). Data are representative of three independent experiments. **(d,f)** Activation status of NF-κB, NFAT, and MAPK signaling or induction of Bcl-x_L_, cyclin D2, and NFATc1/αA in wild-type and *Lrrk1*^*−/−*^ B cells stimulated with anti-IgM, as determined by immunoblot analysis. Data are representative of three independent experiments. **(e)** Nuclear translocation of p65 after stimulation with anti-IgM in wild-type and *Lrrk1*^*−/−*^ B cells assessed by immunoblot analysis. Lamin B and GAPDH serve as nuclear and cytosolic markers, respectively, and as loading controls. Data are representative of three independent experiments. **(g)** Quantitative RT-PCR analysis of Bcl-x_L_ and cyclin D2 expression in B cells 6 h after stimulation with anti-IgM. GAPDH and β-actin were used as internal control for Bcl-x_L_ and cyclin D2, respectively. Data are presented as means ± s.e.m. of two independent experiments. NS, not significant. *P < 0.05, **P < 0.01 (two-tailed unpaired Student’s *t*-test).

Next, we sought to characterize potential signaling defects in *Lrrk1*^*−/−*^ B cells. Because LRRK1 has been reported to regulate endosomal trafficking of EGFR by binding to Grb2^17^, and BCR endocytosis also requires Grb2^25^, we first tested the effect of LRRK1 on BCR internalization. In both wild-type and *Lrrk1*^*−/−*^ B cells, BCR cross-linking led to a rapid decrease in the level of BCR remaining on the surface within 30 min after BCR stimulation similarly, indicating that LRRK1 is not critical for BCR internalization (Fig. 5a). We next examined the activation of various intracellular BCR signaling cascades in wild-type and *Lrrk1*^*−/−*^ B cells. Tyrosine phosphorylation status of Syk and PLCγ2 did not differ significantly between wild-type and *Lrrk1*^*−/−*^ B cells (Fig. 5b). In addition, the absence of LRRK1 did not affect the BCR-induced increase in intracellular Ca^2+^ in total B cell, which is mediated by PLCγ2 (Fig. 5c). This Ca^2+^ increase is larger in MZ B cells than in FO B cells^26^. Given that *Lrrk1*^*−/−*^ mice have a higher percentage of MZ B cells than wild-type mice, we tested Ca^2+^ mobilization separately in MZ and FO B cells in order to accurately evaluate the Ca^2+^ response. LRRK1 did not contribute to Ca^2+^ flux in either subset (Fig. 5c). Taken together, these observations suggest that BCR-mediated proximal signaling is not significantly impaired in *Lrrk1*^*−/−*^ B cells.

BCR stimulation activates the NF-κB, NFAT, and MAPK pathways, which regulate B-cell proliferation and antibody responses. Therefore, we performed immunoblotting to examine the activation status of these signaling molecules. Following BCR stimulation, wild-type and *Lrrk1*^*−/−*^ B cells exhibited similar hallmarks of pathway activation, such as phosphorylation of Erk and dephosphorylation of NFATc2 (Fig. 5d). By contrast, phosphorylation of the NF-κB inhibitor IκBα was significantly reduced in *Lrrk1*^*−/−*^ B cells, accompanied by impaired degradation of IκBα (Fig. 5d). LRRK1 deficiency, however, didn’t affect the NF-κB activation induced by LPS or anti-CD40 stimulation (Supplementary Fig. S3a,b). Furthermore, BCR-mediated nuclear translocation of the NF-κB subunit p65 was much lower in *Lrrk1*^*−/−*^ B cells (Fig. 5e). These data demonstrate that LRRK1 positively regulates NF-κB activation during BCR signaling.

Next, we explored the transcriptional basis for the defect in *Lrrk1*^*−/−*^ B cells. NF-κB signaling drives expression of Bcl-x_L_^27^ and cyclin D2^28^, which respectively control cellular survival and proliferation. Indeed, BCR-stimulated wild-type B cells robustly upregurated Bcl-x_L_ and cyclin D2 proteins, whereas *Lrrk1*^*−/−*^ B cells did not (Fig. 5f). Quantitative RT-PCR analysis showed that synthesis of mRNA encoding these proteins was also considerably impaired in the absence of LRRK1 (Fig. 5g). Given that Bcl-x_L_ and cyclin D2 are required for B-cell survival and proliferation, these results may explain the defect in BCR-activated *Lrrk1*^*−/−*^ B cells. NFATc1/αA, a short isoform of NFATc1, is induced in response to BCR stimulation through the coordinated activation of NF-κB and NFAT signaling^29^. Knockout of NFATc1/αA impairs B-cell proliferation and survival upon BCR stimulation, as well as induction of IgG3 class-switching by TI-2 antigens^30^. BCR-dependent NFATc1/αA expression was considerably impaired in the absence of LRRK1 (Fig. 5f). Collectively, these data suggest that LRRK1 plays a key role in driving the expression of Bcl-x_L_, cyclin D2, and NFATc1/αA following BCR stimulation, which may be downstream effectors of NF-κB.

### LRRK1 synergizes with CARMA1 in regulation of NF-κB activation

**Figure 6:**
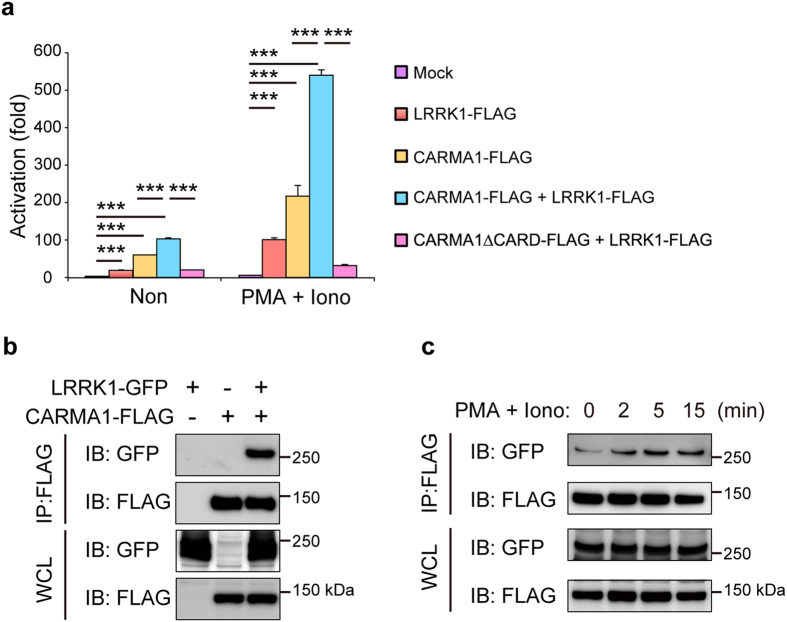
LRRK1 synergizes with CARMA1 in regulation of NF-κB activation. **(a)** Luciferase assay of NF-κB in HEK293T cells transfected with the indicated vectors, and then left untreated (Non) or stimulated with PMA and ionomycin (PMA + Iono). Data are displayed as means ± s.d. of the triplicates. **(b,c)** Binding assays to detect interaction between LRRK1 and CARMA1. GFP-tagged LRRK1, FLAG-tagged CARMA1, or both were transfected into HEK293T cells and stimulated with PMA and ionomycin for the indicated times, and lysates were immunoprecipitated (IP) with anti-FLAG antibody. Anti-GFP or anti-FLAG antibody was used for detection. IB and WCL denote immunoblotting and whole-cell lysate, respectively. Data are representative of three independent experiments. ***P < 0.001 (two-tailed unpaired Student’s *t*-test).

We next asked how LRRK1 links the BCR to NF-κB. Despite their normal response to LPS *in vivo* and *in vitro* (Figs 2a and 4a), *Lrrk1*^*−/−*^ mice have a defect in the antibody response to TI-2 antigens (Fig. 2b), which is mediated primarily through the BCR. In addition, LRRK1 deficiency causes impairment of NF-κB activation upon BCR engagement, but not upon LPS stimulation (Supplementary Fig. S3a). Therefore, we hypothesized that LRRK1 may act on NF-κB signaling components that function downstream of BCR, but not Toll-like receptor 4 (TLR4, a receptor for LPS). We focused on CARMA1 because it is essential for NF-κB activation mediated by BCR, but not TLR4, although this is somewhat controversial^31^,^32^,^33^,^34^,^35^. To test this idea, we first performed luciferase assays to investigate whether LRRK1 functionally associates with CARMA1 to activate NF-κB. As previously reported^36^, we observed that expression of CARMA1 in HEK293T cells resulted in induction of NF-κB activation, which was enhanced by stimulation with phorbol myristate acetate (PMA) plus ionomycin, agents that mimic the effect of BCR stimulation (Fig. 6a). Expression of CARMA1 together with LRRK1 dramatically enhanced NF-κB–driven luciferase activity, particularly when the cells were stimulated with PMA and ionomycin, whereas LRRK1 expression alone had a relatively small effect. The CARD domain of CARMA1 is indispensable for the interaction with BCL10 and subsequent NF-κB activation pathway^7^. Coexpression of LRRK1 and CARMA1 lacking the CARD domain failed to increase NF-κB activity (Fig. 6a), suggesting that LRRK1-mediated NF-κB activation requires CARMA1. Notably, LRRK1 kinase activity was not necessary for this effect: in the presence of CARMA1, kinase-dead LRRK1 enhanced NF-κB activity as effectively as the wild-type protein (Supplementary Fig. S4).

Furthermore, co-immunoprecipitation assays in HEK293T expressing GFP-tagged LRRK1 and FLAG-tagged CARMA1 revealed that LRRK1 physically interacted with CARMA1 (Fig. 6b). This interaction was significantly enhanced when cells were stimulated with PMA and ionomycin (Fig. 6c). BCL10, which is also essential for BCR-activated NF-κB signaling, participates in LPS-mediated NF-κB activation in B cells. However, we detected no physical association between LRRK1-GFP and BCL10 fused to the V5 epitope (data not shown). Together, these findings suggest that LRRK1 positively regulates CARMA1-dependent NF-κB activation, potentially through a physical association between LRRK1 and CRAMA1.

## Discussion

In this study, we discovered that LRRK1 plays a critical role in B-cell development and antibody production by regulating NF-κB signaling. *Lrrk1*^*−/−*^ mice exhibited impaired development of peritoneal B1a cells and were unable to produce IgG3 antibody following TI-2 stimulation due to reduced GLT and AID expression. *In vitro* analysis revealed that LRRK1 is required for BCR-mediated B-cell proliferation and survival. In mechanistic terms, LRRK1 deficiency caused the interruption of the NF-κB signaling pathway and expression of its downstream target genes in response to BCR ligation. Moreover, our findings that LRRK1 interacts with CARMA1 and positively modulates CARMA1-dependent NF-κB activation provide clues about how LRRK1 contributes to NF-κB signaling.

B1a-cell development is controlled by the strength of the BCR signals; genetically engineered mice with weak BCR signals often exhibit reduced numbers of B1a cells, whereas augmentation of the BCR signal frequently increases the B1a-cell population^37^,^38^. Our finding that *Lrrk1*^*−/−*^ mice had a severe defect in B1a-cell development suggests that LRRK1 plays a key role in the BCR signaling pathway. Indeed, we observed that B cells lacking LRRK1 exhibited impaired BCR-induced NF-κB activation. These data are consistent with multiple previous reports that ablation of NF-κB signaling components, including p50, c-Rel, CARMA1, BCL10, and MALT1, in B cells resulted in a block in B1a-cell development^12^,^39^. However, these proteins are also essential for MZ B-cell development, *Lrrk1*^*−/−*^ mice possessed a sufficient number of MZ B cells. Of note, some BCR-proximal signaling components that function in signal transduction pathway upstream of NF-κB, such as BLNK^40^,^41^, BCAP^42^, Btk^43^, PLCγ2^44^, PKC-β^43^, and CIN85^45^, contribute to the development of B1a cells, but not MZ B cells. It has been proposed that in the absence of these molecules, the resultant weakened BCR signals might be sufficient to generate MZ B cells, but not B1 cells^43^. In the context of this model, it seems likely that LRRK1 serves as a key mediator of strong BCR signals that are essential for positive selection of B1a cells.

B1 and MZ B cells participate jointly in the early response to TI-2 antigens by generating IgM and IgG3 antibodies, although their relative contributions vary in an antigen-specific manner: in the case of hapten-Ficoll, MZ B cells are the predominant responders^46^. In this study, *Lrrk1*^*−/−*^ mice failed to mount antigen-specific IgG3 response when immunized with NP-Ficoll, whereas IgM production was largely intact. Given the dispensability of LRRK1 for MZ B-cell development, LRRK1 may be required to transduce BCR signals leading to induction of CSR for IgG3 production. Although the principal BCR-mediated signal that elicits CSR to IgG3 has not been fully elucidated, it is known that NF-κB takes part in this event. The NF-κB complex formed in BCR-activated B cells is primarily composed of the subunits p50, p65 and c-Rel; in the absence of these molecules B cells express markedly reduced levels of germ-line Cγ3 RNA, and consequently produce less IgG3 following appropriate stimulation^47^,^48^,^49^,^50^. Furthermore, the NF-κB pathway initiates and sustains transcription of *AID*^51^. Consistent with this, B cells from *Lrrk1*^*−/−*^ mice immunized with NP-Ficoll exhibited a profound decrease in AID expression. Therefore, our findings regarding the impact of LRRK1 on NF-κB–mediated AID and GLT expression provide molecular evidence that LRRK1 contributes to TI-2–dependent IgG3 antibody production.

Although defects in NF-κB signaling are often associated with inhibition of both IgM and IgG3 production in the TI-2 response^52^, LRRK1 deficiency only impaired IgG3 production. This could be explained by two mechanisms. First, because *Lrrk1*^*−/−*^ B cells exhibited only a partial defect in NF-κB activation, LRRK1 may not be the sole regulator of NF-κB, and quantitative and qualitative dependency on LRRK1 differs between NP-specific IgM and IgG3 production. Second, LRRK1 may regulate TI-2 responses through an alternative pathway such as IFN-γ. IFN-γ stimulates IgG3 production by promoting class switching to IgG3 by acting directly on anti-δ-dex-activated B cells *in vitro*^53^, and immunization with TNP-Ficoll induces IFN-γ production *in vivo*^54^. Given that *Lrrk1* mRNA was upregulated after stimulation with IFN-γ (data not shown), the defects in IgG3 production in *Lrrk1*^*−/−*^ mice might be partially due to an IFN-γ mediated pathway.

We found that *Lrrk1*^*−/−*^ B cells exhibited defective proliferation and survival following BCR stimulation. However, neither the LPS- nor CD40-mediated response was affected by deletion of LRRK1, suggesting that LRRK1 plays a key role in activation of signaling through BCR, but not through LPS or CD40. Importantly, the defects in *Lrrk1*^*−/−*^ B cells correlated with the inability to induce expression of cyclin D2 and Bcl-x_L_ upon BCR stimulation. Furthermore, cyclin D2-deficient mice have reduced numbers of B1a cells, supporting the *in vivo* relevance of LRRK1-mediated up-regulation of cyclin D2. Although previous studies showed that Bcl-x_L_ plays an important role in survival of germinal center B cells and the subsequent TD response, *Lrrk1*^*−/−*^ mice had no effect on the TD response. Given that *Lrrk1*^*−/−*^ B cells responded normally to CD40 stimulation, CD40-mediated expression of Bcl-x_L_ could compensate for the loss of Bcl-x_L_ induction by BCR activation. The normal TD response in *Lrrk1*^*−/−*^ mice is also supported by our observation that *in vitro* co-stimulation of anti-IgM with IL-4 or anti-CD40 could greatly restore impaired BCR-induced proliferation in *Lrrk1*^*−/−*^ B cells. In addition to cyclin D2 and Bcl-x_L_, our data indicated that expression of NFATc1/αA was also reduced in *Lrrk1*^*−/−*^ B cells upon BCR stimulation. Notably, ablation of NFATc1/αA, a prominent short isoform of NFATc1, in B cells caused defects in BCR-mediated proliferation and IgG3 CSR upon immunization with TI-2 antigen, resulting in a phenotype very similar to that of *Lrrk1*^*−/−*^ mice^30^. Therefore, it is tempting to speculate that LRRK1 plays a role in B-cell responses via NFATc1/αA activity. Although NFATc1/αA expression induced by BCR is controlled by NFAT and NF-κB^29^, deletion of LRRK1 did not affect NFATc2 activation, potentially accounting for the contribution of LRRK1-dependent NF-κB signaling.

Among the various signaling transduction pathways we tested, including BCR internalization, Ca^2+^ flux, Syk, PLCγ2, NFATc2, NF-κB, and Erk, only NF-κB activation was specifically impaired in *Lrrk1*^*−/−*^ B cells upon BCR stimulation. Our findings that B cells lacking LRRK1 were hyporesponsive to BCR, but not to LPS or CD40, lead us to propose that LRRK1-dependent regulation of NF-κB activation is largely specific to BCR-mediated signaling. Assembly of an NF-κB activating platform containing CARMA1, BCL10, and MALT1 requires BCR-dependent NF-κB activation. CARMA1 functions as a scaffold platform, and its regulatory mechanism has been extensively investigated by the identification of CARMA1-binding proteins including CaMK II, PKC-β, PKCθ, PKC-δ, HPK1, IKKβ, and CK1α^12^,^55^. These protein kinases can phosphorylate CARMA1 to positively or negatively regulate its activation status. In this study, we identified LRRK1 as a novel binding partner of CARMA1. LRRK1 synergizes with CARMA1 to enhance NF-κB activity. However, this effect is independent of LRRK1 kinase activity, because a kinase-dead version of LRRK1 also enhanced NF-κB–driven luciferase activity in HEK293T cells (Supplementary Fig. S4). Instead, because LRRK1 contains multiple protein-protein interaction domains and regulates the function of binding proteins such as STAM1 in a kinase-independent manner^17^, it seems likely that LRRK1 influences NF-κB signaling via binding to CARMA1. As we also noted that LRRK1 expression alone in HEK293T cells slightly but significantly increased the NF-κB activation, it may be possible that LRRK1 would regulate endogeneous CARMA3 or other NF-κB signaling components as well as CARMA1. Further studies are needed to determine how LRRK1 activates NF-κB.

There is growing evidence that dysregulation of NF-κB signaling contributes to various lymphoid malignancies. IKK-dependent activation of NF-κB is a potential downstream target of BCR-ABL1^56^, which interacts with LRRK1. In BCR-ABL1–positive cells, attenuation of LRRK1 function by overexpression of a dominant-negative form makes cells more susceptible to DNA damage induced by the anticancer drug, etoposide^22^. LRRK1 might also regulate survival and proliferation of tumor cells, based on the observation that overexpression of LRRK1 allows cells to proliferate under serum-deprived conditions^21^. Moreover, BCR target genes are upregulated in various human lymphomas, suggesting that therapeutic inhibition of oncogenic BCR signaling might have clinical value^11^. Our findings showing that LRRK1 activates the BCR-dependent NF-κB pathway imply that LRRK1 is involved in tumorigenesis, a possibility that merits further investigation.

## Methods

### Mice, B-cell isolation, and cell culture

C57BL/6J mice were purchased from CLEA Japan (Japan). *Lrrk1*^*−/−*^ mice were described previously^19^. Mice were bred and maintained under specific pathogen–free conditions and used at 8–12 weeks of age. Animal care and experiments were approved by the animal committee of Osaka University, and all experiments were conducted according to the approved guidelines.

For B-cell isolation, resting B cells were purified from spleen by negative selection with anti-CD43 magnetic beads or positive selection with anti-CD19 magnetic beads (Miltenyi Biotech). The purified B-cell population was > 95% positive for B220. B cells were cultured in RPMI supplemented with 10% FCS, 2-ME, penicillin (100 U/ml, GIBCO), and streptomycin (100 μg/ml, GIBCO).

### Flow cytometry analysis

Cells prepared from spleen and peritoneal cavity were stained with the following antibodies: fluorescein isothiocyanate (FITC)-conjugated anti-B220 (RA3–6B2), anti-CD11b (M1/70), anti-CD21 (7G6), anti-CD23 (B3B4), and anti-IgG3 (R40–82); phycoerythrin (PE)-conjugated anti-CD5 (53–7.3), anti-CD23 (B3B4), anti-IgM (II/41), and anti-AA4.1 (AA4.1); allophycocyanin (APC)-conjugated anti-B220 (RA-6B2), anti-IgM (II/41), anti-AA4.1 (AA4.1), and anti-NIP-BSA; phycoerythrin-indotricarbocyanine (PE-Cy7)-conjugated anti-B220 (RA3–6B2), and anti-CD23 (B3B4); and Pacific Blue–conjugated B220 (RA3–6B2). Stained cells were analyzed on a FACS Canto II or LSRII flow cytometer (BD Biosciences), and data were processed using the FlowJo software (TreeStar).

### Immunization and enzyme-linked immunosorbent assay (ELISA)

Mice were injected intraperitoneally (i.p.) with 100 μg of NP-CGG in alum, 50 μg of NP-LPS (Biosearch Technologies), or 50 μg NP-Ficoll (Biosearch Technologies). For secondary immunization, mice were injected i.p. with 50 μg NP-CGG without adjuvant. Levels of NP-specific antibodies were analyzed on 96-well plates coated with 2 μg/ml NP(29)-BSA (Biosearch Technology) and detected with goat anti-mouse IgG and HRP-conjugated anti-goat IgG antibodies (SouthernBiotech). To determine basal immunoglobulin titers from naive mice, sera were quantified by ELISA with HRP-conjugated goat anti-mouse IgM, IgG1, IgG2b, IgG2c, IgG3, and IgA (SouthernBiotech).

### Proliferation and survival assay

Isolated B cells were labeled with 20 μM 5- (and 6-) carboxyfluorescein diacetate succinimidyl ester (CFSE; Life Technologies) at room temperature for 5 min, and then stimulated with LPS (Sigma-Aldrich), anti-CD40 (HM40–3; BD Biosciences), anti-IgM F(ab)’_2_ (Jackson Immunoresearch), or recombinant mouse IL-4 (R&D) for 72 h. Cells were stained with 7-AAD (Invitrogen), and the percentages of viable 7-AAD- and CFSE-diluted B cells were assessed using an LSRII flow cytometer (BD Biosciences). Apoptotic cells were identified using an AnnexinV-APC apoptosis detection kit (BD Pharmingen).

### Quantitative RT-PCR analysis

CD19-positive B cells were collected from spleen to analyze the expression of Iμ-Cγ3, Iμ-Cμ, Iγ3-Cγ3, and AID, and CD43-negative B cells were collected to analyze the expression of Bcl-x_L_ and cyclin D2. For measurement of *Lrrk1* mRNA expression, B-cell populations were sorted on a FACSAria (BD Biosciences). RNA was isolated and purified using the RNeasy kit (Qiagen). cDNA was generated using the PrimeScript RT reagent Kit (Perfect Real Time) (TAKARA). Real-time PCR was performed on a LightCycler96 (Roche) or an ABI Prism 7700 Sequence Detection System (Applied Biosystems). The following primer pairs were used: *Gapdh* (Mm99999915_g1) and *Lrrk1* (Mm00713303) (both from Applied Biosystems); Iμ-Cγ3, sense 5′-CTCGGTGGCTTTGAAGGAAC-3′ and antisense 5′-ACCAAGGGATAGACAGATGGGG-3′; Iμ-Cμ, sense 5′-ACCTGGGAATGTATGGTTGTGGCTT-3′ and antisense 5′-TCTGAACCTTCAAGGATGCTCTTG-3′; Iγ3-Cγ3, sense 5′-AACTACTGCTACCACCACCACCAG-3′ and antisense 5′-ACCAAGGGATAGACAGATGGGG-3′; *Aid*, sense 5′-TGCTACGTGGTGAAGAGGAG-3′ and antisense 5′-TCCCAGTCTGAGATGTAGCG-3′; *Bcl2l1*, sense 5′-GACAAGGAGATGCAGGTATTGG-3′ and antisense 5′-TCCCGTAGAGATCCACAAAAGT-3′; *Ccnd2*, sense 5′-CTGCAGAACCTGTTGACCATCG-3′ and antisense 5′-TAATTCATGGCCAGAGGAAAG-3′; *Gapdh*, sense 5′-CTCATGACCACAGTCCATGC-3′ and antisense 5′-CACATTGGGGGTAGGAACAC-3′; and β*-actin*, sense 5′-AGTGTGACGTTGACATCCGTA-3′ and antisense 5′-GCCAGAGCAGTAATCTCCTTC T-3′.

### BCR internalization

BCR endocytosis was monitored as described previously^57^ with several modifications. In brief, purified B cells were incubated with biotin-labeled anti-mouse IgM F(ab)’_2_ for 30 min on ice. After two washes with ice-cold PBS(-), the cells were chased at 37 °C for the indicated times. The cells were fixed with 1% paraformaldehyde, and biotinylated antibodies remaining on the surface were detected with APC-streptavidin and analyzed by flow cytometry.

### Ca^2+^ measurement

Cytosolic Ca^2+^ concentrations were measured as described previously^58^. Briefly, splenocytes were loaded with indo-1 acetoxylmethylester (Indo-1 AM) in the presence of Pluronic F-127 (Invitrogen) for 45 min. Then cells were stained with antibodies against B220, CD21, and CD23 and stimulated with 10 μg/ml anti-mouse IgM F(ab)’_2_ in 2 mM Ca^2+^. The increase in free intracellular Ca^2+^ was monitored on an LSRII flow cytometer.

### Western blotting and co-immunoprecipitation

CD43-negative B cells (5 × 10^6^ cells) were stimulated with 10 μg/ml anti-mouse IgM F(ab)’_2_, 10 μg/ml LPS, or 2.5 μg/ml anti-CD40 for the indicated times, and then lysed in lysis buffer (10 mM Tris-HCl [pH 7.4], 150 mM NaCl, 1% [vol/vol] Triton X-100, 0.5 mM EDTA) plus protease inhibitor (Roche) and phosphatase inhibitor (Nacalai Tesque) cocktails. Nuclear fractions were prepared by NE-PER Nuclear and Cytoplasmic Extraction Reagent (PIERCE Biotechnology). Samples were transferred to polyvinyldifluoride membranes by electrophoresis and antibodies against the following proteins were used for immunodetection: antibodies against p-Syk (Tyr525/526) (C87C1), Syk, p-PLCγ2 (Tyr1217), phospho-p44/42 MAPK (Erk1/2) (Thr202/Tyr204), p44/42 MAPK (Erk1/2), NFATc2 (D43B1), phospho-IκBα (Ser32/36) (5A5), IκBα, Bcl-xL (54H6), GFP, and HRP-conjugated β-Actin (13E5) were obtained from Cell Signaling Technology; antibody against GAPDH (6C5) was obtained from Millipore; and antibodies against PLCγ2 (Q-20), NFκB p65 (C-20), Lamin B (C-20), cyclin D2 (M-20), and NFATc1 (7A6) were obtained from Santa Cruz Biotechnology. For co-immunoprecipitation, HEK293T cells transfected with CARMA1-FLAG and LRRK1-GFP vector were stimulated with 50 ng/ml PMA and 0.5 μM ionomycin for 0, 2, 5, or 15 min and lysed in lysis buffer containing 0.2% (vol/vol) Nonidet P-40. The CARMA1-FLAG protein was immunoprecipitated with protein G magnetic Dynabeads bound to anti-FLAG (M2) antibody (Invitrogen).

### Plasmid construction, transient transfection, and luciferase assay

FLAG- or GFP-tagged mouse LRRK1 and FLAG-tagged mouse kinase-dead LRRK1 (D1386A) constructs were generated as previously described^19^. The C terminally FLAG-tagged mouse CARMA1 and CARMA1ΔCARD (ΔCARD: amino acids 1–150) fragments were amplified by PCR and cloned into pApuro-IRES-GFP vector^59^. For transient expression, HEK293T cells were transfected with the indicated constructs using FuGENE HD (Promega). For luciferase assays, HEK293T cells were transfected for 24 h with renilla luciferase plasmid (E6921) (Promega), NF-κB firefly luciferase vector (Addgene), and the indicated expression vectors using FuGENE HD, and then stimulated with 50 ng/ml PMA and 0.5 μM ionomycin for 12 h. Activity was measured using the Dual-luciferase Reporter System (Promega) and normalized to renilla luciferase activity used as an internal transfection control.

### Statistical analysis

Statistical analyses were performed by two-tailed unpaired Student’s *t* test. Differences with *P* values < 0.05 were considered significant. *P < 0.05; **P < 0.01; and ***P < 0.001.

## Additional Information

**How to cite this article**: Morimoto, K. *et al.* LRRK1 is critical in the regulation of B-cell responses and CARMA1-dependent NF-κB activation. *Sci. Rep.* **6**, 25738; doi: 10.1038/srep25738 (2016).

## Supplementary Material

Supplementary Information
