# Supplementary material for: LRRK1 is critical in the regulation of B-cell responses and CARMA1-dependent NF-κB activation
Source: Sci Rep. 2016 May 11;6:25738. doi: 10.1038/srep25738 (PMC4863158; doi:10.1038/srep25738)
Supplement: Supplementary Information [file srep25738-s1.pdf]

Supplementary Information

LRRK1 is critical in the regulation of B-cell responses and CARMA1-dependent NF-κB activation

Keiko Morimoto, Yoshihiro Baba, Hisaaki Shinohara, Sujin Kang, Satoshi Nojima, Tetsuya Kimura, Daisuke Ito, Yuji Yoshida, Yohei Maeda, Hana Sarashina-Kida, Masayuki Nishide, Takashi Hosokawa, Yasuhiro Kato, Yoshitomo Hayama, Yuhei Kinehara, Tatsusada Okuno, Hyota Takamatsu, Toru Hirano, Yoshihito Shima, Masashi Narazaki, Tomohiro Kurosaki, Toshihiko Toyofuku & Atsushi Kumanogoh

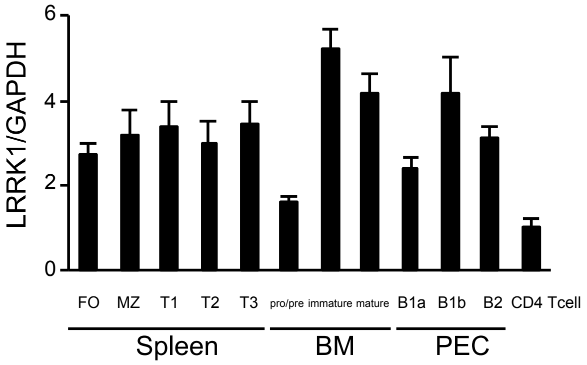

Supplementary Figure S1. LRRK1 mRNA expression in B-cell subsets. Quantitative RT-PCR of *Lrrk1* mRNA in B-cell subsets, normalized to the corresponding level of *Gapdh* mRNA. B-cell subsets were gated as follow: spleen, follicular (FO; $B220^{+}CD21^{lo}CD23^{hi}$), marginal zone (MZ; $B220^{+}CD21^{hi}CD23^{lo}$), T1 ($IgM^{hi}CD23^{-}AA4.1^{+}B220^{+}$), T2 ($IgM^{hi}CD23^{+}AA4.1^{+}B220^{+}$), and T3 ($IgM^{lo}CD23^{+}AA4.1^{+}B220^{+}$); bone marrow (BM), pro/pre ($IgM^{-}B220^{lo}$), immature ($IgM^{+}B220^{lo}$), and mature ($IgM^{+}B220^{hi}$); peritoneal cavity (PEC), B1a ($IgM^{+}CD5^{+}$), B1b ($IgM^{+}CD5^{-}CD11b^{+}$), and B2 ($IgM^{+}CD5^{-}CD11b^{-}$). Data are presented as means ± s.d.

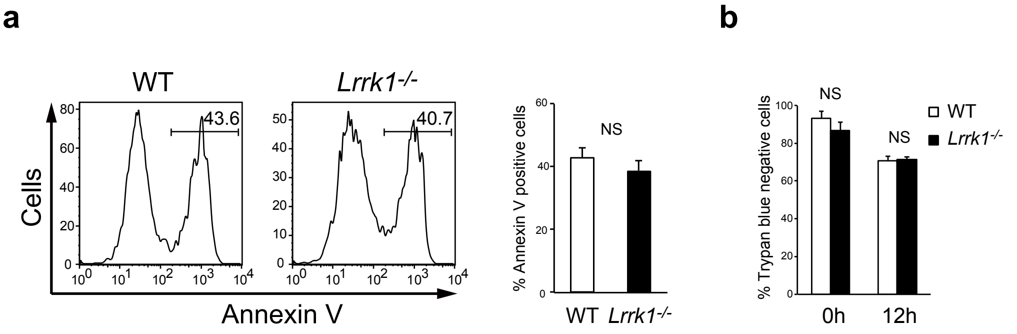

Supplementary Figure S2. The intact survival rate of unstimulated *Lrrk1*$^{-/-}$ B cells. **(a)** Flow cytometry of Annexin V staining of wild-type and *Lrrk1*$^{-/-}$ B cells after 12 h culture without stimulation. **(b)** Cell viability was determined by Trypan blue staining in (a). Data are presented as means ± s.d. for 3-4 mice. NS, not significant. (two-tailed unpaired Student's *t*-test)

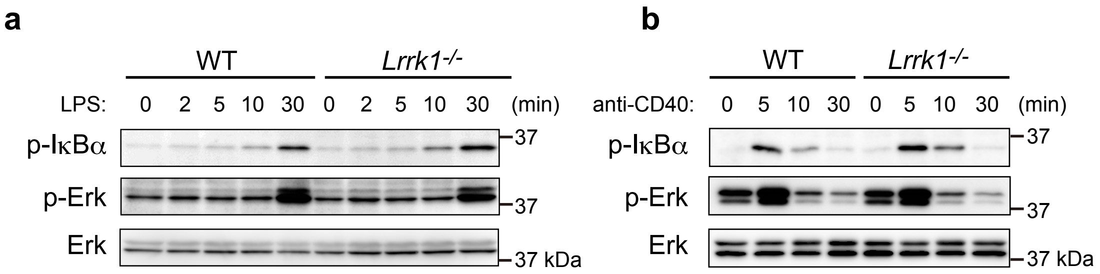

Supplementary Figure S3. Normal NF-κB signaling upon LPS or anti-CD40 stimulation in *Lrrk1*$^{-/-}$ B cells. (a,b) Immunoblot analysis of whole-cell lysates of wild-type and *Lrrk1*$^{-/-}$ B cells stimulated with 10 μg/ml LPS (a) or 2.5 μg/ml anti-CD40 (b) for the indicated time periods. Data are representative of at least two similar experiments.

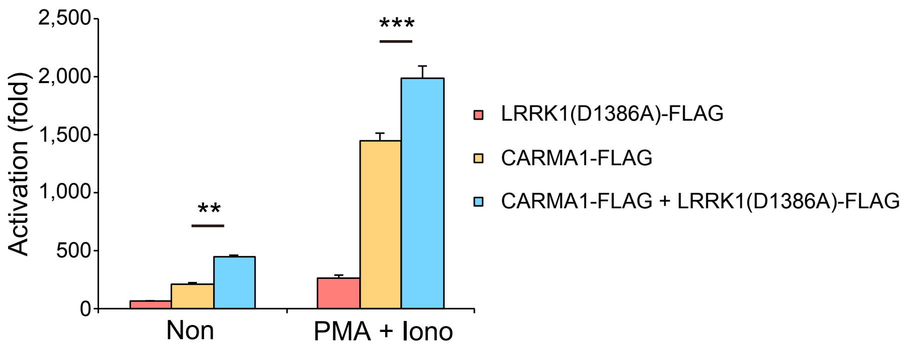

Supplementary Figure S4. CARMA1-mediated NF-κB signaling is independent of LRRK1 kinase activity. Luciferase assay of NF-κB–driven transcription in HEK293T cells transfected with a kinase dead LRRK1 (LRRK1 (D1386A) -FLAG) and/or CARMA1-FLAG, and then left untreated (Non) or stimulated with PMA and ionomycin (PMA + Iono) for 12 h. Data are displayed as means ± s.d. of triplicates. $^{**}P < 0.01$, $^{***}P < 0.001$ (two-tailed unpaired Student's *t*-test)

/ B cell (%)	WT	*Lrrk1*$^{-/-}$
pro/pre	52.1 ± 4.7	57.9 ± 1.6
immature	14.7 ± 2.2	16.0 ± 1.0
mature	33.2 ± 6.9	26.1 ± 2.2

Supplementary Table S1. B-cell development in bone marrow of *Lrrk1*$^{-/-}$ mice. Percentages of cells in B-cell subsets in bone marrow samples from wild-type and *Lrrk1*$^{-/-}$ mice. B-cell subsets were gated as follow: pro/pre ($IgM^{-}B220^{lo}$), immature ($IgM^{+}B220^{lo}$), and mature ($IgM^{+}B220^{hi}$). Data are presented as means ± s.e.m. of three independent experiments.
